# The subjective career success of women: The role of personal resources

**DOI:** 10.3389/fpsyg.2023.1121989

**Published:** 2023-03-28

**Authors:** Eileen Koekemoer, Chantal Olckers, Pieter Schaap

**Affiliations:** Department of Human Resource Management, Faculty of Economic and Management Sciences, University of Pretoria, Pretoria, South Africa

**Keywords:** job demands resources model, mediation, moderation, social cognitive theory, person job fit (PJ-fit), person environment fit, resilience, grit

## Abstract

**Introduction:**

Research on women’s career success has been the subject of extensive investigations, emphasizing the barriers they encounter in their careers. However, far less attention has been given to the personal resources that promote women’s career success. The purpose of our study was to provide more conclusive evidence regarding the role that personal resources such as resilience and grit can play in the relationship between women’s person-environment fit and the perceptions of their career success. Underpinned by the Job Demands Resources and social cognitive theory, our study aims to investigate whether resilience and grit could either explain how person-environment fit translates into feelings of subjective career success or could strengthen this relationship.

**Method:**

A cross-sectional online survey research design was used, and a convenience sample of 408 female employees was obtained. Relationships were explored through structural equation modelling.

**Results:**

When controlling for age, the findings of this study revealed significantly positive relationships between the constructs, with person-environment fit, resilience, and grit, explaining a large portion of the variance in subjective career success. Although our data supported the mediating role of grit and resilience in the person-environment fit and subjective career success relationship, the moderating effects of grit and resilience could not be established.

**Discussion:**

These findings illustrate both grit and resilience as mechanisms that indirectly affect the person-environment fit and subjective career success relationship of women. However, our findings indicate that resilience and grit cannot be considered mechanisms that would buffer against poor person-environment fit’s effect on their career success perceptions. Firstly, our study advances our understanding of the roles personal resources such as resilience and grit play in women’s career success as ways to overcome obstacles and workplace barriers. Secondly, using the motivational process of the Job Demands Resources Framework as theoretical background, we contribute by shedding light on how personal resources (resilience and grit) can be considered underlying factors influencing the person-environment fit and career success relationship for women. If women experience good person-environment fit, there is a greater opportunity for developing resilience and grit and, consequently, subjective career success.

## Introduction

Across the globe, women’s entering the workforce has remained steady. According to the ILO (International Labour Organisation, 2022), the female labour force participation rate worldwide was 46% in 2021 (International Labour Organisation, 2022). As more women enter and remain employed, a growing scholarly interest in studying women’s careers is evident (Cadaret et al., 2017). Although female professional careers only began to be mainstreamed in the last century (Younger et al., 2015), according to Khilji and Pumroy (2019), women’s career advancement has received a fair share of interest in the literature. However, entering the workforce did not guarantee an easy path for women, and barriers to career success and advancement are well documented in recent literature (Ibáñez, 2017; Kim and O’Brien, 2018; Schizas et al., 2022). Scholars agree that there are different and more challenges for career women (Carli, 2020), as they have to navigate their attention between work and housework, are confronted with fears of adverse performance evaluations, and face a higher risk of job loss and increased threats of violence, exploitation, and harassment (Leskinen et al., 2015; Mittal and Singh, 2020).

However, despite being subjected to imposed traditional gender roles, patriarchal culture, gender stereotypes, differential treatment, and male egos in their workplace (Leskinen et al., 2015; Cadaret et al., 2017), some women are rising to the highest levels in their organisations (Carboni et al., 2020), and employers see the value that professional women bring to the workplace. Research on the career success of women from specific cultures or in certain professions has been the subject of extensive investigations (Cadaret et al., 2017; Ibáñez, 2017; Kim and O’Brien, 2018; Schizas et al., 2022), with a strong emphasis on the problems/barriers they encounter in their careers. However, far less attention has been given to factors or personal resources that promote women’s career success. According to Younger et al. (2015), evidence relating to how women strengthen their internal resources to sustain their career goals and increase their career persistence is lacking.

According to Bandura’s (1986) social cognitive theory, personal, behavioral, and environmental variables affect career performance. To this end, in the study of Carvalho et al. (2018), women emphasised their agency and active role in career-making. Furthermore, they placed the key to their career success at the individual level, where specific individual enablers such as effort, hard work, dedication, competence, and desire to seize challenges lead to intrinsic satisfaction. This resonates with the notion of Savickas and Mark (2002) that people possess resources that they employ to exploit the opportunities available in an external environment (i.e., career construction theory). In the literature, personal resources have been defined as traits (e.g., grit and personality), states (e.g., hope and optimism), or mental abilities (e.g., resilience) people use to increase the control they have over their environment and to improve their perceived fit between person and job (Bakker and Demerouti, 2017).

Similarly, the fit of person and environment (person-environment fit) has been indicated as a dominant force in explaining job satisfaction, performance, and career success (Ballout, 2007). As a result, person-environment fit has received considerable scholarly interest, with the main findings illustrating that fit matters and leads to positive career outcomes (Su et al., 2015). While the relationship between person-environment fit and career success has been established (Xi et al., 2022), scholars have advocated for more research toward understanding the psychological processes or factors underlying fit and the fit-outcome relationships (Su et al., 2015).

Incidentally, the availability of psychological resources such as grit and resilience for women’s career success has been investigated (Carboni et al., 2020). Resilience is known to help individuals to progress in their careers by providing them with the capacity to overcome obstacles, learn, and grow (Kuntz et al., 2016). Specifically, for women, it has been argued that resilience could serve as a barrier to alleviate the adverse effects of work stress caused due to the prejudice of the glass ceiling, thus, strengthening their ability to break the glass ceiling in organisations (Tabassum et al., 2019). In this regard, senior women managers exemplify resilience, as it helps them to navigate complexities and obstacles amidst the challenges and hardships they experienced in their careers (Jogulu and Franken, 2022). Women seem to believe that even with obstacles, they can fight for their right to promotion and career advancement. As a result, their positive attitude toward career advancement has been referred to as resilience (Smith et al., 2012). According to Tabassum et al. (2019), for women, resilience is a process of moving forward after significant setbacks and is developed over time.

Moreover, Salisu et al. (2020) suggest that individuals who are resolute and better capable of enduring, adapting, and building up their strengths are more likely to succeed in their careers. In the context of the numerous obstacles women face, this requires employing the resources they possess and adapting to their circumstances to advance or be successful in their careers. Career success has been described as a journey with a long-term orientation (Visagie and Koekemoer, 2014) and necessitates determination and endurance to attain long-term career goals (Popoola and Karadas, 2022). In this regard, having a motivating attitude of passion and perseverance towards long-term goals despite setbacks, i.e., displaying grit (Duckworth et al., 2007), is believed to be a significant personality trait for career success and performance, even more so for women (Popoola and Karadas, 2022). The concept of grit is well-defined in the literature and is the ability to stay focused and dedicated to long-term goals, despite obstacles or adversity (Duckworth and Quinn, 2009). Grit within the context of career success has been considered an essential element since grittier employees use their competencies better as they are less focused on short-term goals and less influenced by setbacks or failures, and are better able to confront challenging situations (Salisu et al., 2020). Studies investigating grit among women are on the rise, as they often list grit as a personal attribute when asked to describe the traits that led to their success. Several studies found grit as a common trait shared among women leaders, where strong correlations with their career advancement or success (Popoola and Karadas, 2022) are evident. Overall, scholars seem to agree that grit enables women leaders to persevere in facing the many hurdles and barriers they encounter in their professional career trajectories.

In essence, the literature shows that overcoming workplace barriers may be essential to women’s career growth or success, and understanding the interplay between person and environment is crucial. Regarding drivers of subjective career success, personality traits and job resources are considered essential as people can use them to choose and shape their working environment. Aligned with the social cognitive theory, understanding how well women persevere in the workplace and the level of success they attain despite obstacles are major elements worthy of more research. In this regard, we argue that for women’s career success, it is important to consider person-environment fit and the influence and use of their personal resources or traits (i.e., grit and resilience) as ways to overcome obstacles and workplace barriers. In this regard, Carstens et al. (2021), have illustrated the role of resilience as a moderator in the relationship between person-environment fit and subjective career success amongst a sample of males and females. Considering the important notion that grit involves long-term perseverance, understanding the role of grit for women’s career success may add valuable insight into the stamina women display to keep on moving forward to achieve their career goals. Understanding how women respond to setbacks in the short term (i.e., by being resilient), and stay focused and dedicated in achieving their long-term career goals despite obstacles and adversity in their careers (having grit), may add to the body of knowledge on women’s career development. Given the empirical evidence that grit and resilience are relatively distinct constructs in positive psychology (Meyer et al., 2020), examining their role in the person-environment fit and career success relationship seems particularly pertinent.

## Literature review and hypotheses development

### Person-environment fit and career success

Within the field of vocational psychology, the person-environment fit is considered a dominant research topic as it suggests that behavior can be understood based on the fit between an individual and their environment. In essence, the person-environment fit is described as the compatibility (or fit) between individuals (employees) and their work (organisation) (Kristof, 1996), to such an extent that higher fit perceptions significantly improve employees’ work attitudes, their intention to remain at the organisation, greater levels of job satisfaction, job engagement, organisational commitment, and lower turnover (Oh et al., 2014). Overall, when there is a good fit between employees and their organisation or job, employees are likely to engage in development-seeking behaviours and create situations that support higher levels of job performance and achievement (Ballout, 2007). Indeed, it is believed that person-environment fit significantly interacts with individual and situational variables for explaining individual employability and career success (Ballout, 2007). This is because individuals are attracted to and seek out career opportunities with organisations where they believe they will fit in and be able to realize their career ambitions.

Furthermore, employees will develop a promising career and achieve professional success if the value of individuals fit with their organisation is conducive to career development (Guan et al., 2021). Thus, central to the person-environment-fit theory are employees’ perceptions about the extent to which their work setting facilitates or hinders their personal development and growth (Guan et al., 2021). Overall, fit literature suggests that when individuals acquire abilities and skills that meet the job requirements, obtain resources that meet their needs, and experience value congruence with their organizations, they are more likely to generate positive attitudes, behaviors, and performance (Kristof-Brown, 2000; Cable and DeRue, 2002).

Several conceptualisations (supplementary and complementary fit) and commonly investigated types of fit (e.g., person-vocation fit, person-organization fit, and person-job fit) that differentially relate to employee attitudes and behaviors are found in the literature. In this regard, Greguras and Diefendorff (2009) contend that different types of fit may satisfy different psychological needs and that the satisfaction of different psychological needs relates to distinct employee outcomes. Xi et al. (2022) recently found that demands-abilities fit, needs-supplies fit, and person-organisation fit is strongly correlated with indicators of employees’ subjective career success (career satisfaction) and objective career success (mobility and promotability). Demands-ability fit and the needs-supply fit are usually referred to as complementary fit as one entity (either the person or the work setting) provides something that the other one wants. For instance, employees may possess specific skills or knowledge an organisation seek, or the organisation may offer the type of resources or rewards that the person requires or desire. As a result, a good fit is perceived (De Cooman and Vleugels, 2022). For career scholars, the importance of person-environment fit and implications for employees’ objective and subjective career success has long been argued (Kristof-Brown et al., 2005).

Career success is generally referred to as “work-related accomplishments/outcomes that individuals achieve through their work experience over time” (Seibert and Kraimer, 2001, p. 2). It is categorised as having two distinctive dimensions. An objective component refers to aspects such as salary, pay and promotion, and a subjective component refers to perceived career achievement and career satisfaction (Salisu et al., 2020). Nowadays, objective evaluations of career success seem to play a lesser role in the contemporary career landscape characterised by boundaryless and protean careers. In this landscape, individuals, rather than their employing organisations, become the architects of their careers and development, and they take responsibility for managing their careers and transforming their career paths. It seems that how personally meaningful careers are and how individuals experience their career success have become important (Ng and Feldman, 2014). This is also true in the case of women, as scholars argue that the career success of professional women is not entirely an objective construct (Adapa and Sheridan, 2021). In this regard, Krishnan et al. (2020) postulates that the subjective perceptions of career success cause an increase in the motivation and performance of women managers. For the purpose of this study, we conceptualised person-environment fit according to the definition of Cable and DeRue (2002), comprising of three dimensions: Person-organisation fit, Need-supply fit, and Demand-ability fit. Given the context above, the following hypothesis is set forward:

*H1*: Person-environment fit positively influences women’s subjective career success.

### Resilience and subjective career success

Against the backdrop of person-environment fit, it has been argued that person and environment are not stable entities and, as a result, (as with career success) should be managed over time (Kim et al., 2020). In this sense, De Cooman and Vleugels (2022) argued that when a person and their work environment are no longer congruent, they will seek to change their characteristics. This implies the need to rely on individual characteristics or resources to obtain fit or to increase positive outcomes. This aligns with the context of protean careers, where employees are taking a more proactive role in managing their careers. Therefore, the development of personal-career-related capabilities and dispositions is significant. According to Converse et al. (2012), employees will use such capabilities to effectively influence their career environment and regulate their behaviour to succeed in more volatile work settings. In this regard, resilience is put forward to help employees manage the ever-changing situations and setbacks experienced at work. Overall, resilience is the ability of employees to bounce back from adverse situations or successfully adapt to negative situations (Wagnild, 2009). The need for women to be resilient has become more evident with the overwhelming amount of literature focusing on the barriers and challenges they face in their careers. It might explain their success against all odds (Salisu et al., 2020). Overall, resilience is a behavioural capability that reflects resource utilization and the ability to adapt continually at work (Kuntz et al., 2016). According to Kuntz et al. (2017. p. 421), resilience “comprises adaptive, proactive, support-seeking, learning, and crisis management behaviours that can be continually developed and enacted in everyday practice.” This is in line with Guillén (2020), that postulates that it is essential for individuals who want to be successful in their careers to remain attentive under pressure and not to be disheartened by career setbacks as situations where resilient behaviours are needed are inevitable.

Career scholars agree that career success involves achieving long-term career goals; resilience has been linked to goal striving and attainment (Salisu et al., 2020). Salisu et al. (2020) state that no successful person succeeds without experiencing and overcoming significant challenges; therefore, resilience is considered the differentiating factor between those succeeding and those less successful. Rodríguez-Sánchez and Perea (2015) also postulate that successful employees can endure and turn out strong after harsh conditions and hardship.

Several studies have shown resilience as a personal factor contributing to career success (Wei and Taormina, 2014; Salisu et al., 2020). According to Han et al. (2021), resilient individuals are more likely to show adjustment behaviours associated with perseverance (i.e., not giving up in the face of career setbacks). In addition, they are more resourceful and determined to overcome barriers. Because resilient individuals are also known for their high levels of persistence and adaptability, they are considered better equipped to overcome career obstacles and disruptions (e.g., barriers to achieving career goals, uncertainty, and poor relationships with co-workers). Given the previous description of resilience, we propose the following hypothesis:

*H2:* Resilience positively influences women’s subjective career success.

### Grit and subjective career success

An employee who wants to achieve career-related goals must possess a personal characteristic, such as grit, that can serve as a personal resource (Van Zyl et al., 2022). Grit is about having a long-term passion for one’s selected goal and staying dedicated to achieving such a goal (Duckworth et al., 2007). It is about steadfastly holding on to a goal even when the road is bumpy and progress toward the goal is slow (Duckworth and Quinn, 2009). Grit is a non-cognitive psychological construct and signature strength (Clark and Clark, 2019) that comprises two components: consistency of interest and perseverance of effort. *Consistency of interest* relates to the tendency of individuals to continuously re-engage with and remain focused on specific tasks and goals over time. It is about having a passion defined as an intense enthusiasm to stick to a target for an extended period (such as building a successful career). *Perseverance of effort* relates to the tendency to work hard to achieve set goals despite setbacks, failures, or stumbling blocks (Duckworth and Quinn, 2009). Grit, thus, influence an individual’s attitude and behaviour toward long-term goals (Fosnacht et al., 2018) and has been related to various aspects in the literature, such as longevity in the workplace and marriage (Eskreis-Winkler et al., 2014), commitment to a career (Credé, 2018), successful aging in the elderly (Kim and Lee, 2015), higher levels of engagement (Azari Noughabi et al., 2022).

Although grit interacts with the individual’s potential and predicts academic achievement, performance, and success, Duckworth et al. (2007) argued that grit, not intelligence, is the most reliable predictor of personal success and performance. This is because of gritty individuals’ ability to use their strengths to complete tasks and invest sustained energy in achieving their goals, despite setbacks (Duckworth, 2016). Furthermore, evidence that grit predicts success in various domains, such as academic success, educational attainment, effectiveness, training completion, and task performance, is found in the literature (Duckworth et al., 2007, 2011; Eskreis-Winkler et al., 2014; Van Zyl et al., 2022).

The role of grit within the career success context is supported by social cognitive career theory (Lent and Brown, 2019). The main elements of social cognitive career theory focus on the individual’s perseverance in their work environment and the level of success they attain despite the setbacks and obstacles they may experience. Grit requires hard work to overcome challenges and maintain interest and effort over a long period, despite failure, obstacles, and plateaus in progress (Duckworth et al., 2007). Even though signs of boredom and disappointment might be experienced in their career path, the gritty individual adjusts trajectory to stay on course (Duckworth et al., 2007). In this regard, grit’s definition of passion and perseverance in attaining long-term goals despite obstacles align with the focus of perseverance in the working environment despite setbacks, as depicted in the social cognitive career theory (Lent and Brown, 2019).

Subjective career success is more than just an ultimate destination; it is about a lifelong journey toward achieving goals (Visagie and Koekemoer, 2014). Given this definition and the inclination of grit being a long-term orientation towards achieving goals, it seems plausible that if an individual applies grit in working towards long-term work-related goals, feelings of perceived subjective career success will be experienced (Olckers and Koekemoer, 2021). Furthermore, aligned with the notion that career success is an ongoing process aimed at achieving career-related goals, gritty individuals or employees are more likely to persevere in performing their duties at work as they keep an eye on longer-term career goals (Kabat-Farr et al., 2019), and as such may be more successful in their careers.

Clark and Clark (2019) emphasised the importance of including career success in grit studies because, from the positive psychology viewpoint, careers provide individuals with a sense of identity and meaning. However, only a limited number of studies have investigated this relationship (Eskreis-Winkler et al., 2014; Credé, 2018; Clark and Clark, 2019). Credé’s (2018) research suggested that gritty individuals achieve greater career success and are more likely to succeed in achieving their personal goals. Eskreis-Winkler et al. (2014) also confirmed an association between grit and career success, where workplace retention was used as a proxy for career success. Although the quantitative data of Clark and Clark (2019) could not establish a linear relationship between grit and career success, in their qualitative data, participants considered grit essential to their career success and the foundation upon which career success builds.

Popoola and Karadas (2022) recently found that female employees (in security forces) displaying high levels of grit tend to be subjectively successful in their careers. In this regard, these authors also argued that grit is a sufficient resource to achieve subjective career success. Salisu et al. (2020) also argued that grittier entrepreneurs persevere when faced with difficulty and can uphold their pursuit of perplexing long-term goals such as career success. Furthermore, they found that the perseverance of effort dimension of grit positively relates to three components of subjective career success (i.e., career satisfaction, perceived career achievement, and perceived financial attainment). On the other hand, the consistency of interest dimension was positively related to only perceived financial attainment and appeared to be a less significant predictor of career satisfaction and perceived career achievement. Danner et al.’s (2020) study measured career success by both an objective (income) and subjective (job satisfaction) dimension. It confirmed grit as a stronger predictor of subjective career success than objective career success, which aligns with the findings of Ion et al. (2017). Grit has also been confirmed as a personal attribute related to female educational leaders’ career success and advancement (Hogan and Larkin-Wong, 2013). Overall, these studies corroborate the relevance of grit for women’s career success. Therefore, the following hypothesis is formulated:

*H3*: Grit positively influences women’s subjective career success.

### The role of resilience and grit as personal resources

In this study, the role of personal resources (i.e., resilience and grit) in the person-environment fit and subjective career success relationship is explored and explained using the Job Demands-Resources (JDR) model of Xanthopoulou et al. (2007) and the Conservation of Resources (COR) theory (Hobfoll, 1989). According to the JDR framework, the well-being and performance of employees (which could also refer to the subjective career success of women) are influenced by their work conditions comprising both job demands and resources (Demerouti et al., 2001). *Job demands* refer to any negatively perceived physical, psychological, social, or organisational aspects of the job that require continuous effort and skills from the employee that consumes their energy. *Job resources*, however, are any physical, psychological, social, or organisational aspects of the job that serve as a motivational and functional component to employees resulting in achieving organisational goals, positive attitudes at work and encouraging personal growth and development (e.g., career opportunities, social support, task identity, person-environment fit) (Bakker and Demerouti, 2017).

In general, the notion is that job demands and resources impact the performance of individuals and organisations through a dual process, i.e., either a health impairment or a motivational process. The *health impairment process* confers that high job demands or poorly designed jobs lead to the exhaustion of resources, which will increase stress and job strain, negatively impacting employees’ well-being and performance. In contrast, the availability of job resources activates the *motivational process* of the JDR. If employees possess the necessary job resources to fulfil their job tasks, it will motivate them to perform. In addition, the motivational process will buffer the impact of high and harmful job demands on the well-being and performance of employees (Teuber et al., 2021). This motivational process of the JDR is used in this study to explain how personal resources (i.e., resilience and grit) can be utilised to facilitate the person-environment fit and subjective career success relationship of women. Bakker and Demerouti (2017) argued that personal resources play a similar role as job resources within the motivational process of the JDR. Therefore, these personal resources could potentially fulfil three different roles by (1) directly effecting the outcome variable (i.e., subjective career success); (2) indirectly effect the relationship between the job resource (i.e., person-environment fit) and the outcome variable, and (3) moderate or magnify the effect job resources have on subjective career success. In line with the aforementioned, we discuss the role of resilience and grit (as personal resources in the JDR model) in the person-environment fit and career success relationship hereafter.

### The role of resilience in the person-environment fit and career success relationship

Within the context of women’s careers, we view resilience as a personal resource, an inherent trait that enables women to cope with obstacles and adversity in their careers. Against the backdrop of resilience as a personal resource, some scholars have proposed the moderating and mediating role of emotional resilience (Li et al., 2020). Jaureguizar et al. (2018) specifically refer to a protective model of resilience, where the buffering effect of resilience in the stress mental health relationship was investigated. In line with the person-environment fit theory, researchers have postulated that resilience is not only about the individual but also about the environment in which the individual functions and how employees can respond to adversity or personal setbacks in their work roles (Rutter, 2007). In this regard, Crane and Searle (2016) argued that resilience is not just about individual-level capacities but an interaction between individual characteristics and opportunities for resilience building allowed by the environment. In this sense, they argued that the work environment influences resilience positively and negatively, which can include both challenge and hindrance stressors. They specifically found evidence of the mediating role of resilience in the stressor-strain relationship where challenges and hindrances (as stressors) respectively enhance or diminish resilience with consequent influences on the strain of employees. Overall, supporting the notion that challenge stressors may create opportunities for the capacity building of resilience, in contrast to hindrance stressors that deplete the capacity for resilience. In line with such findings and the context of the JDR model and person-environment fit, Caniels et al. (2022) similarly found that subjective person-environment fit, induces employees to behave resiliently.

Consequently, individuals with high trait resilience behave more resilient at work because of the high person-environment fit they experience (i.e., they are likely to perceive more resources in their environment). Thus, within the context of the career success of women, it stands to reason that being resilient, when faced with career challenges and adversities (which is very common for women’s careers), will likely result in persistence in achieving career goals which will lead to the satisfaction of one’s career progression. This is because resilient individuals are more deliberate in assessing their strengths and weaknesses and seek opportunities to upgrade their skills to respond quickly to possible career disruptions (Mishra and McDonald, 2017).

Furthermore, Han et al. (2021) argue that due to their stronger capacity to adapt to the demands of their job and cope with stress, resilient employees may be less subject to suffering negative consequences of work stress. Against the backdrop of the motivation process of the JDR model, they postulate that because resilient career employees are more ambitious and motivated to advance in their careers, they may encounter more stressful situations in their work lives but may also be better equipped to cope with this stress effectively, as they are more adaptable and confident in their abilities. Overall, the literature has already demonstrated the vital role of resilience in the relationship between stress and mental health as a mediator and a moderator (Li et al., 2020). Similarly, we argue that the personal resource of resilience can either magnify (moderate) the person-environment fit career success relationship of women or explain (mediate) how perceptions of person-environment fit translate into subjective career success for women. In this regard, we propose the following two hypotheses:

*H4a*: Resilience moderates the relationship between person-environment fit and women’s subjective career success.

*H4b*: Resilience mediates the relationship between person-environment fit and women’s subjective career success.

### The role of grit in the person-environment fit and career success relationship

According to the person-environment fit theory, person-environment fit indicates alignment between what the individual is passionate about, what they are competent at, what they like to do, and the nature of their job (De Cooman and Vleugels, 2022). Thus, there is a deep connection between an individual’s values, passions, and work environment, the feeling that they can contribute their knowledge and skills to something they value (demands-ability fit), and the rewards they can receive for their service (needs-supply fit). If this deep connection is present, it will most probably result in a deeper, more personal connection with one’s work (thus implying person-job fit) and organisational goals, with the result that individuals will be more inclined to show sustained effort and perseverance in achieving these job or career goals despite the challenges and setbacks they might be facing (Van Zyl et al., 2022). This is supported by Peterson and Seligman (2004), who stated that individuals are more likely to use their personal strengths at work if there is congruence between their characteristics and that of their job or organisation. Thus, when women use their strengths at work, it will foster a deeper interest in obtaining their career-related goals, and therefore, they will show more perseverance in achieving them. Women will be more likely to invest effort and persevere with the challenges they face in building their careers if they experience deep congruence between their personal interests and values and the nature of the work environment or requirements of their jobs (Van Zyl et al., 2022). A significant positive relationship between the interest and perseverance components of grit and good person-environment fit was confirmed by Van Zyl et al. (2022). Furthermore, several studies have confirmed a positive relationship between person-environment fit and subjective career success (Ballout, 2007; Salisu et al., 2020; Xi et al., 2022) and between grit and subjective career success (Danner et al., 2020; Salisu et al., 2020; Popoola and Karadas, 2022). There thus seems to be theoretical support to suggest that grit indirectly affects (mediate) the relationship between person-environment fit and subjective career success.

However, the moderating effect of grit, as personal resource, to this established person-environment fit and career success relationship has received minimal attention. Grit’s role as a potential moderator could perhaps explain how different levels of person-environment fit (as a job resource) can influence women’s perceptions of their career success. In this sense, we propose that women who experience congruence between their personal characteristics, needs, and abilities and the requirements of their jobs and who are passionate and have the desire to build a successful career will most likely be more successful in their career compared to those employees who display lower levels of grit (Duckworth et al., 2007). Thus, the presence of grit will strengthen the relationship between person-environment fit and subjective career success. In addition, grit could also serve as a buffer against poor person-environment fit’s negative effect on women’s perceptions of career success (Van Zyl et al., 2022). Women will thus invest in and utilise their grit to compensate for the negative impact poor person-environment fit can have on achieving their career-related goals (Del Castillo, 2020). We, therefore, postulate that if women are grittier and faced with challenging work situations such as poor person-environment fit, they will still be able to build successful careers. De Cooman and Vleugels (2022) argue that aligning an individual’s signature strengths and job requirements can strengthen the person-environment fit and success relationship. In the presence of poor person-environment fit, and line with COR theory, women will most likely activate their personal resources (i.e., grit) to deal with mis-fit and to avoid it having a negative impact on their careers.

Based on the discussion above, we formulate the following two hypotheses:

*H5a*: Grit moderates the relationship between person-environment fit and women’s subjective career success.

*H5b*: Grit mediates the relationship between person-environment fit and women’s subjective career success.

Thus, based on the literature above, our study aims to determine the role of both resilience and grit as personal resources in the relationship between person-job fit and subjective career success.

## Materials and methods

### Sample and procedure

The targeted group for this study was employed women with a minimum of at least one year of working experience. A cross-sectional online survey research design was used, and a convenience sample of 408 female employees was obtained. Data was collected over a four-month period in 2020 (commencing in January 2020, prior to the hard lockdown of the global Covid-19 pandemic). The majority of the sample was White (79,4%), Afrikaans-speaking women (65,7%) with less than 5 years’ work experience (49,75%). More than halve of the sample (56,1%) is 40 years and younger. The majority (71, 4%) of the female participants were well educated and had completed tertiary education. Of the sample, 34% operated on middle and senior managerial levels in their respective organisations. Table 1 depicts the demographic information obtained from the biographical questionnaire.

**Table 1:** Demographic profile of the sample (*N* = 408).

Variable	Grouping	Frequency	Percentage
Age (years)	20–30	116	28.4
	31–40	113	27.7
	41–50	68	16.7
	51–60	85	20.8
	60+	26	6.4
Ethnicity	White	324	79.4
	African	49	12
	Coloured	20	4.9
	Indian	13	3.2
	Other	2	0.5
Home Language	Afrikaans	268	65.7
	English	90	22.1
	African languages	47	11.52
	Other	3	0.7
Educational level	Grade 12	117	28.7
	Bachelor’s degree	120	29.4
	Honours degree	115	28.2
	Master’s degree	50	12.3
	Doctoral degree	6	1.5
Years of work experience	Less than 2	91	22.3
	2–4 years	114	27.9
	5–7 years	63	15.4
	8–10 years	38	9.3
	11–13 years	28	6.9
	14–16 years	13	3.2
	17+ years	52	12.7
	Missing values	9	2.2
Level	Operational	120	29.40
	Junior management	53	13
	Middle management	78	19.10
	Senior management	62	15.20
	Executive	28	6.80
	Missing values	67	16.42

Missing values within the demographics have been accounted for.

Inviting participants were requested *via* email to complete the survey *via* a secure Qualtrics web-link. Informed consent was obtained from each participant, who completed the survey voluntarily and anonymously. Ethical approval to conduct the study was obtained from the relevant tertiary institution.

### Measures

The 9-item *Person-Environment Fit Scale* developed by Cable and DeRue (2002) measured person-environment fit. This scale comprises three dimensions, each measured by three items: Person-organisation fit, Need-supply fit, and Demand-ability fit. In addition, a 7-point Likert-type scale ranging from 1 (Strongly disagree) to 7 (Strongly agree) is used. Hinkle and Choi (2009) reported acceptable Cronbach’s alpha coefficients for the three sub-dimensions: Person-organisation fit (*α* = 0.98), Need-supply fit (*α* = 0.96), and Demands-ability fit (*α* = 94).

The *Subjective Career Success Inventory* developed by Shockley et al. (2016), comprising 24 items with eight dimensions (Recognition, Quality work, Meaningful work, Influence, Authenticity, Personal life, Growth and development, and Satisfaction), was used to measure subjective career success. Each dimension was measured by three items scored on a 5-point Likert-type scale ranging from 1 (not at all) to 5 (a great deal). Shockley et al. (2016) reported a Cronbach’s alpha value of 0.94 for the overall scale, with values ranging between 0.77 and 0.92 for the dimensions.

Resilience was measured by the 14-item *Resilience scale* developed by Wagnild (2009) and scored on a 7-point Likert-type scale ranging from 1(strongly disagree) to 7 (strongly agree). Acceptable Cronbach’s alpha values ranging between 0.84–0.91 was reported for the scale (Wagnild, 2009).

The *Grit-S* developed by Duckworth and Quinn (2009) was used for measuring grit and comprises eight items measured by two dimensions: Consistency of interest (reversed scored) and perseverance of effort. Each dimension was measured by four items scored on a 5-point Likert scale ranging from 1 (not at all like me) to 5 (very much like me). In addition, acceptable Cronbach’s alphas ranging from 0.73 to 0.83 have been reported for the overall scale (Von Culin et al., 2014; Gonzalez et al., 2019).

A biographical questionnaire was also administered, requesting the female participants to report on their age, ethnicity group, home language, educational level, and tenure. We control for age since grit is believed to increase with age (Pena and Duckworth, 2018).

### Data analyses

This study used Mplus statistical software version 8 (Muthén and Muthén, 2017) for all analyses. We used a two-stage approach in the analyses. Firstly, confirmatory factor analyses (CFA) were used to confirm the measurement models that allowed for a single score. Using a single factor score for a measure in a Structural Equation Model (SEM) regression model has practical advantages such as avoiding multicollinearity, model identification, and convergence problems when testing complex structural models. Secondly, to avoid biased estimates in the SEM model due to measurement error, plausible values representing latent factor scores were generated using multiple imputation procedures and the Bayes estimator (Asparouhov and Muthén, 2010) (The procedure is commonly used for handling missing values in data). We used MPlus Markov chain MC Bayesian estimation utilities with Gibbs sampler (PX1) and 50,000 iterations to impute 30 PV datasets (Muthén and Muthén, 2017). Convergence was monitored with a potential scale reduction (PSR) indicator (PSR should be below 1.05) and trace plots. Mplus’s default diffuse priors’ settings were used, and data were combined using Rubin’s (1987) method.

We tested for measurement models that allowed for univocal scoring and represented the common variance that underlay the items of the measure were theoretically justified. Rodriguez et al. (2016a) have shown that most self-report measures of psychological constructs that are presented as multidimensional consist of a substantive general or common factor. After partially out of the general factor in multidimensional models, the sub-factors rarely are unique and substantive. Therefore, all the measures used in this study were tested for the presence of a substantive common factor, also known as essential unidimensionality, for scoring. Reise et al. (2010) argue that an essential unidimensional measurement model represents a single common construct where the effect of observed multidimensionality is attributed to negligible parcels of similar content or non-substantive facets. Non-substantive facets of a broader construct manifest as correlated residuals or shared uniqueness between items in a unidimensional factor model and may adversely effect model fit when items are numerous. The residuals were allowed to correlate in a measurement model if they were justified and were done sparingly.

To test for essentially unidimensionality supported by theory, we used CFA and tested for one-factor models. We employed maximum likelihood as estimator (ML) to estimate model parameters and model-fit. We used Kline’s (2016) suggested goodness-of-fit indices and cut-off points determine model fit: (1) absolute fit indices: the root mean square error of approximation (RMSEA <0.08), and the standardized root mean square residual (SRMR <0.08); (2) incremental fit indices: the Tucker-Lewis index (TLI > 0.90), and the comparative fit index (CFI > 0.90). In addition, as was suggested by McNeish et al. (2018), we also assessed measurement quality by inspecting the standardised loadings ( λ > 0.40) and item uniqueness (> 0.1 but <0.9) of the observed variables in our models. Bifactor modelling in CFA was used as a technique to test for essential dimensionality for measures consisting of numerous items and acceptable model fit for a strict unidimensional model could not be obtained. Goodness-of-fit indices have proven to be in-affective when testing for essential dimensionality for measures with multiple items (Rodriguez et al., 2016a). Instead, we used Bifactor strength indices to evaluate the plausibility of the data supporting an essentially unidimensional model (Rodriguez et al., 2016b).

We reported the following descriptive statistics: skewness and kurtosis. Univariate and multivariate normality were tested for skewness and kurtosis and were appraised according to the +2/−2 range (Kim, 2013). We employed Mardia’s multivariate normality test to evaluate the normality assumption. We used Mahalanobis distance values and Cook’s distance (gCD*i*) statistics as global indices to test for multivariate outliers (Aguinis et al., 2013). We specifically tested for outliers on the factor scores derived from the measurement model, for these were likely to directly impact the hypothesis tested in the structural models (SEM). These manifest factor scores were obtained using the regression method in Mplus and closely represent the plausible values used in the SEM models for this study. We traced the cause of the identified outliers back to the items used in the measure. Moreover, the manifest factor scores were used to study the distribution characteristics of the data.

To determine the level of reliability of the scales used, McDonald’s omega (> 0.70; Hayes and Coutts, 2020) was estimated. According to Crutzen and Peters (2015), the omega coefficient provides a more accurate approximation of the internal structure of a scale than Cronbach’s alpha. We have used the CFA factor loadings to calculate McDonald’s omega coefficient. We also calculated the factor determinacies scores to determine the correlations among the items with the latent factor which is also an indication of the reliability of the scales and which should preferably be higher than 0.80 (Wang and Wang, 2020). The statistical significance level for the correlation coefficients was set at 95% (*p* ≤ 0.05). Following the guidelines of Ferguson (2009), the practical significance of the correlations was set at 0.10 (small effect), 0.30 (medium effect) and 0.50 (large effect). Satorra and Saris (1985) power analysis for SEM models was used to determine if the sample size was sufficient for detecting statistically significant small effects in the models.

## Results

### Testing the measurement models

The results of the tests for essentially unidimensional models are reported in Table 2. Results for Mardia’s multivariate skewness (*b*_1,2_ = 1033.81, *p* < 0.01) and kurtosis (*b*_2,2_ = 4575.05, *p* < 0.01) for the items used in the measurement models tested were statistically significant, suggesting that the data was not normally distributed. As a result, the Maximum Likelihood Robust (MLR) estimator was used to test the measurement models.

**Table 2:** Fit statistics per measurement model.

Final models	Model fit indices							Factor loadings		
	χ2	*df*	CFI	TLI	RMSEA	90% CI	SRMR	MIN	MEAN	MAX
Person-Job fit	47.82*	8	0.95	0.90	0.11	0.08, 0.14	0.07	0.48	0.70	0.90
Grit	3.341*	2	1.00	0.99	0.04	0.00; 0.11	0.02	0.60	0.62	0.74
Resilience	145.50*	64	0.89	0.87	0.06	0.04; 0.65	0.05	0.39	0.53	0.68
Subjective career success (bi-factor model)	540.89*	228	0.92	0.90	0.06	0.05, 0.06	0.06	0.33	0.56	0.73

χ^2^, chi-square statistic; df, degrees of freedom; CFI, comparative fit index; TLI, Tucker-Lewis index; RMSEA, root mean square error of approximation; CI, confidence intervals; SRMR, standardised root mean square residual.

* *p* < 0.01.

Power for detecting the statistical significance (*p* = 0.05) of a small effect size (0.15) for the measurement model (Subjective career success) with the most degrees of freedom (228) in this study, was sufficient at 0.83 (Satorra and Saris, 1985). The remaining measurement models and SEM models in this study had lower degrees of freedom (from 3 to 64), and all met the power expectations (≥0.80) for detecting the statistical significance of a small effect size (≤0.10). Therefore, it can be concluded the sample size was sufficient for the study.

The CFA results for the one-factor model of the resilience measure (14 items) suggested the data supports an essentially unidimensional model. Only one item from the resilience scale (‘I usually take things in stride) was removed due to a low factor loading (<0.30). Although the CFI and TLI values did not meet the cut-off criteria (>0.90), the SRMR and RMSEA values showed an acceptable model fit. A unidimensional model containing several items with large degrees of freedom hardly ever describes real data and is routinely rejected based on the results of statistical model fit indices (Bentler, 2007). According to Reise et al. (2010), the prospect of finding a perfectly unidimensional model in assessment data is almost zero. Marsh et al. (2013) supported this, who stated that the assumptions of unidimensionality are rarely obtained from the questionnaire data that are so often used for research in the social sciences. One correlated residual was freed for RES14 and RES7 items that both reflect finding a way out of difficulty and the lengthiest in the measure, which may suggest item redundancy and method effects. No more substantive misspecifications on the modification index (expected parameter changes (EPCs) within or close −0.10 to 0.10) could be identified, which suggests the lower-than-ideal CFI and TLI values can be ascribed to multiple non-substantive misspecifications or white noise (Muthén and Asparouhov, 2012).

The essential unidimensional model was not supported for the 9-item Person-Environment Fit Scale due to the items of the person-organization fit not loading significantly on the unidimensional scale. Consequently, only complementary fit (person-job fit) comprising Need-supply fit and Demand-ability fit was included in the analysis. Table 2 suggests an acceptable model fit for the model after the residual variance for two of the person-environment fit items were allowed to correlate due to similarity in item wording (‘My abilities and training are a good fit with the requirements of my job’ with ‘My personal abilities and education provide a good match with the demands that my job places on me’) (Wang and Wang, 2020).

Although the two-factor structure (i.e., perseverance of effort and consistency of interest) of grit has been confirmed in several studies (Li et al., 2018; Schmidt et al., 2018; Van Zyl et al., 2022), other studies measured grit as a unidimensional construct (Ion et al., 2017; Gonzalez et al., 2019). Like the procedure followed by Vazsonyi et al. (2019), we modelled grit as a unidimensional construct with latent factors to reflect positively and negatively worded items. However, the model was abandoned for the consistency of interest dimension because it did not provide salient loadings on the common factor. In addition, the method effects of reversed items appeared detrimental to obtaining a single score for the scale. Consequently, only the perseverance of effort scale items was included in the study. Using only the perseverance of effort component is also in line with previous studies. For example, both Bowman et al. (2015) and Danner et al. (2020) concluded that the perseverance component of grit is a stronger predictor of success than the consistency of interest and only measured the perseverance component of grit. Table 2 suggests an acceptable model fit for the scale.

The subjective career success measure was analyzed using a bifactor model after the one-factor model showed an unacceptable model fit. The factor strength indices, namely, the explained common variance (EVC = 0.56), Omega Hierarchical for the general factor (Omega H = 0.866), and for specific factors (OmegaHS range between 0.22 to 0.56), the Percentage of Uncontaminated Correlations (PUC = 0.91) and Factor Determinacy coefficient (FD = 0.944) suggest that measure may be regarded as essentially unidimensional (Rodriguez et al., 2016b). Consequently, we used the general/common factor score in SEM analyses.

The measurement model results for each of the constructs measured are displayed in Table 2. Although we tried to retain as many as possible of the original items for each contract to ensure the psychometrical soundness in the measurement models, we removed items with low factor loadings (<0.30). Since all of the scales have been used previously in other studies, we used a CFA approach and evaluated the original conceptualization of the constructs based on theory. Following the guideline by Jackson et al. (2009), we only made *post hoc* model re-specifications when these modifications were practical or theoretically justifiable to improve model fit. An overview of the measured constructs and the fit indices per construct are displayed in Table 2.

Table 3 presents the descriptive statistics and the reliabilities of the factor scores. The skewness values for the manifest factor scores ranged between the suggested +2 and − 2 values, some of the kurtosis values were above range, suggesting some outliers in the dataset. The reliabilities of all the factor scores were considered good since all McDonald’s omega values ranging between 0.75 and 0.98 were higher than the recommended value of *ω* > 0.70 (Hayes and Coutts, 2020). The factor determinacy values (FD ≥ 0.90; Wang and Wang, 2020) showed strong correlations between the factor scores and the latent factor. Note the difference in the Hierarchal Omega ωh/ωs values for the general (ωh) factor and the specific factors, suggesting the specific factors (ωs) are non-substantive after the common variance in general factor was partialled out.

**Table 3:** Descriptive statistics, reliability and factor determinacy.

Variable	Skewness	Kurtosis	*ω*	FD
**Person-Job fit**	−1.04	1.08	0.92	0.96
**Grit**	−1.28	3.34	0.74	0.86
**Resilience**	−1.22	3.38	0.83	0.92
**Subjective career success** (PUC = 0.91, ECV = 0.56)	−0.74	0.78	0.87^ωh^	0.94
Recognition	−0.98	1.14	0.47^ωs^	0.80
Quality work	−0.93	1.25	0.40^ωs^	0.79
Meaningful work	−0.78	0.68	0.24^ωs^	0.70
Influence	−0.69	0.59	0.26^ωs^	0.73
Authenticity	0.85	0.80	0.22^ωs^	0.68
Personal life	−0.80	0.55	0.56^ωs^	0.86
Growth & development	−0.66	0.19	0.44^ωs^	0.84
Satisfaction	−1.02	1.15	0.28^ωs^	0.77

*ω*, McDonald’s Omega; ωh/ωs Hierarchal Omega; FD, Factor determinacies.

#### Testing for outliers on factor scores

The Mahalanobis distance values and Cook’s distance (gCDi) statistic identified one extreme outlier case in the data that was used for the SEM analysis. The extreme values identified was related to the Grit scale. The case showed extremely low self-ratings of 1 on the 5-point Likert scale for three of the positively scored grit items GR6, GR7, GR8, while item GR5 showed a rating of 3 (all items measure the perseverance component of grit). The preceding negatively scored items GS1 to GS4 (measuring the consistency of interest component of grit that was not included in the final SEM model) all obtained ratings of 2. This inconsistent responding suggest that the respondent may have been confused by the direction of the scales and negatively versus positively worded items. The outlier case can most likely be ascribed to error arising from momentary inattentiveness of the respondent that led to the misresponding. Consequently, the case was excluded from further analyses.

#### Factor scores intercorrelations

The correlations between the disattenuated factor scores (plausible values) derived from the measurement models using multiple imputation procedures and Bayes estimator are presented in Table 4. Converge of the estimation was acceptable with PSR < 1.05, and trace plots displayed clear mixing. These factor scores were used in the SEM models tested in this study. Statistically significant (*p* < 0.05) correlations were found between the variables. Statistically significant relationships were found between person-job fit and both grit (*r* = 0.16; medium effect) and subjective career success (*r* = 0.66; large effect). Similarly, a statistically significant relationship was reported between grit and subjective career success (*r* = 0.60; large effect) and between resilience and subjective career success (*r* = 0.59; large effect). Age did show a statistically significant correlation with the person-environment fit (*r* = 0.13; small effect) and subjective career success (*r* = 0.16; small effect). However, age showed a statistically insignificant correlation with grit (*r* = 0.04; small effect) and resilience (*r* = 0.06; small effect).

**Table 4:** Factor score inter-correlations.

Latent variable	1	2	3	4
1. Person-Job fit	–			
2. Resilience	0.22*	–		
3. Subjective Career Success	0.66*	0.59*	–	
4. Grit	0.17*	0.60*	0.46*	–
5. Age	0.13*	0.06	0.16*	0.04

Disattenuated correlation coefficients (* = statistically significant *p* < 0.05).

### The structural models and hypotheses testing

Maximum likelihood (ML) with bias corrected bootstrapping was used for the SEM models tested in the study. Bootstrapping has proved to be effective in calculating accurate standard errors for parameter estimated on non-normal data.

#### Testing the moderation of resilience

**Figure 1:**
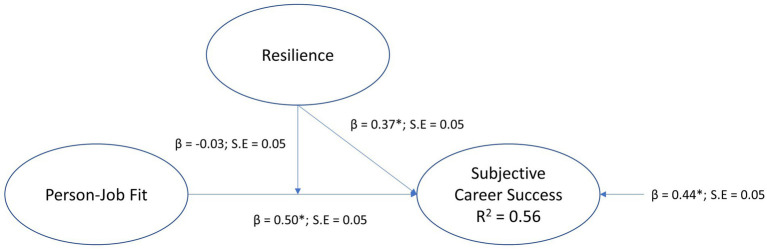
Moderation model for resilience.

The structural path model of the moderation model for resilience, where person-job fit was positioned as the exogenous variable, resilience the moderator, and subjective career success the endogenous (outcome) variable, is depicted in Figure 1.

The model was a just identified model (*χ*2 [0, *N* = 408]; = 0, *p* = 1.0; CFI = 1.00; TLI = 1.00; RMSEA = 0.00 [0.00 0.00]; SRMR = 0.00). Hypothesis H4a was not supported since the results indicate that resilience did not significantly moderate the regression path from person-job fit to subjective career success for the interaction effect (person-job fit x resilience) was statistically insignificant (*β* = −0.03, *p* > 0.05, small effect). Resilience had a statistically significant direct effect on subjective career success (*β* = 0.37, *p* < 0.01, medium effect), supporting hypothesis H2, and person-job fit had a statistically significant direct effect on subjective career success (*β* = 0.50, p < 0.01, large effect), providing support for hypothesis H1. The model explained 56% variance in subjective career success (*R*^2^ = 0.56).

#### Testing the mediation model of resilience

**Figure 2:**
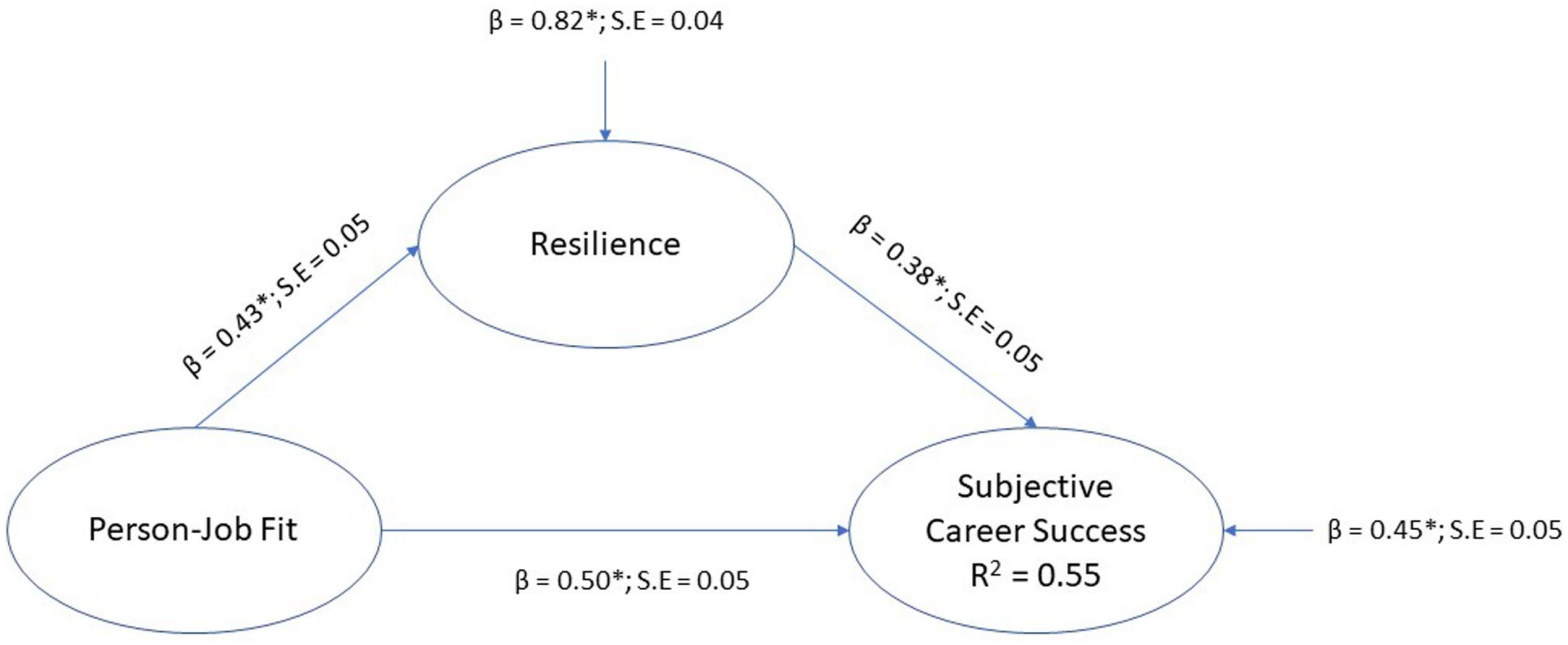
Mediation model for resilience.

The structural path model of the mediation model for resilience, where person-job fit was positioned as the exogenous variable, resilience the mediator, and subjective career success the endogenous (outcome) variable, is depicted in Figure 2.

The model was a just identified model (*χ*2 [0, *N* = 408]; = 0, *p* = 1.0; CFI = 1.00; TLI = 1.00; RMSEA = 0.00 [0.00 0.00]; SRMR = 0.00). The results indicated that resilience significantly mediates the regression path from person-job fit to subjective career success (*β* = 0.16, *p* < 0.01, small effect), therefore, hypothesis H4b was supported. Resilience had a statistically significant direct effect on subjective career success (*β* = 0.38, *p* < 0.01, medium effect), and person-job fit had a statistically significant direct effect on subjective career success (*β* = 0.50, *p* < 0.01, large effect). The model explained 55% variance in subjective career success (*R*^2^ = 0.55).

#### Testing the moderation model of grit

**Figure 3:**
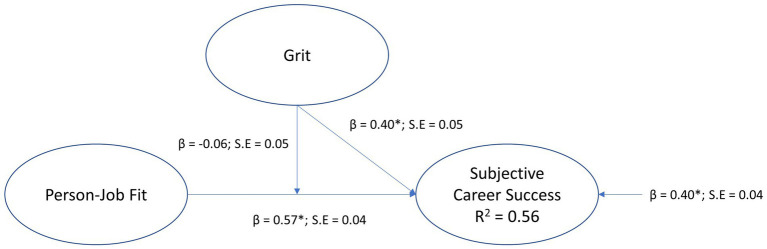
Moderation model for grit.

The structural path model of the moderation model for grit, where person-job fit was positioned as the exogenous variable, grit the moderator, age as covariate for grit, and subjective career success as the endogenous (outcome) variable is depicted in Figure 3.

The model fit indices suggested a model with unacceptable fit (*χ*2 [3, *N* = 407]; = 17.14, *p* < 0.01; CFI = 0.94; TLI = 0.85; RMSEA = 0.108 [0.06 0.16]; SRMR = 0.10) (see Figure 3). The residual covariance matrix showed that age was misspecified and caused the model to show lower model fit. Consequently, age was removed from the model and a just identified model was obtained (*χ*2 [0, *N* = 407]; = 0, *p* = 1.0; CFI = 1.00; TLI = 1.00; RMSEA = 0.00 [0.00 0.00]; SRMR = 0.00). Except for very small differences standard errors (on the third decimal place), the regression paths were unchanged for the model. The results indicate that grit had an insignificant moderating effect on the regression path from person-job fit to subjective career success for the interaction effect (person-job fit x grit) was statistically insignificant (*β* = −0.06, *p* > 0.01, small effect). Grit had a statistically significant direct effect on subjective career success (*β* = 0.40, *p* < 0.01, medium effect) and person-job fit had a statistically significant direct effect on subjective career success (*β* = 0.57, *p* < 0.01, large effect). The model explained 56% variance in subjective career success (*R*^2^ = 0.56). Hypothesis H5a was, therefore, not supported.

#### Testing the mediation model of grit

**Figure 4:**
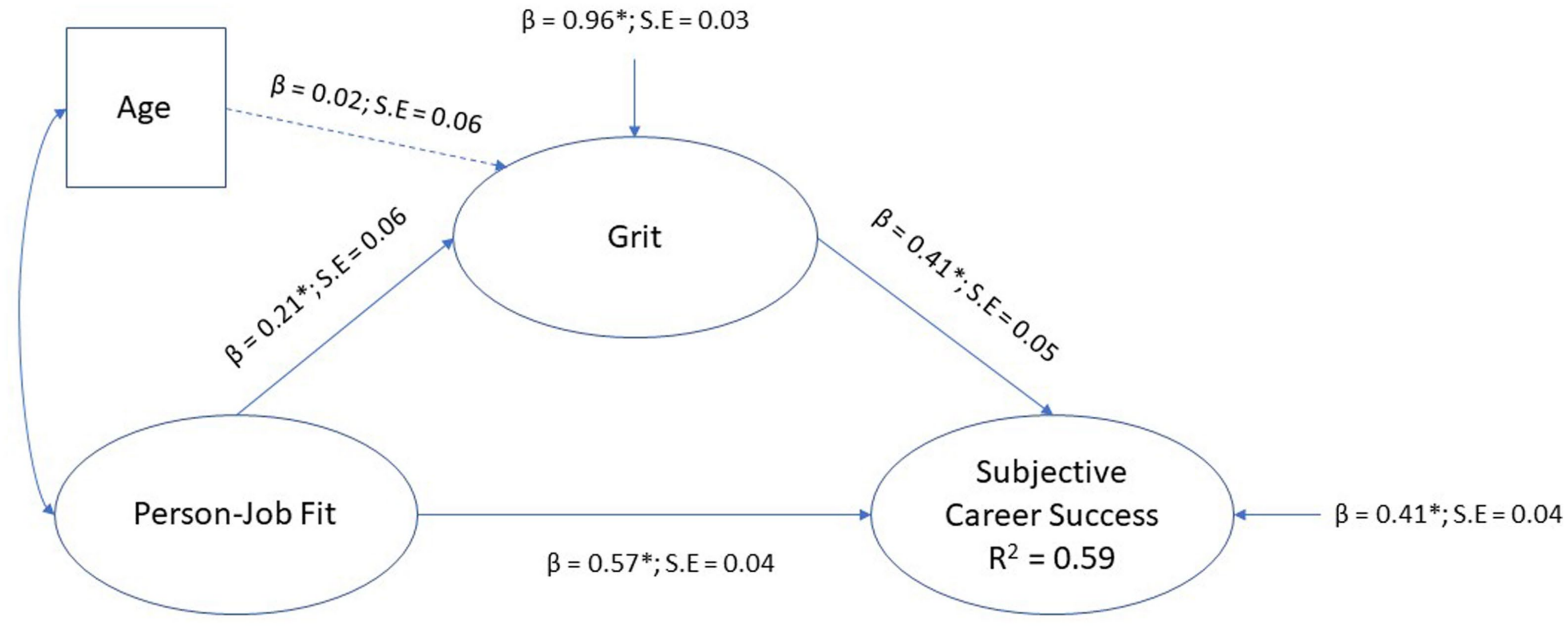
Mediation model for grit.

The structural path model of the mediation model for grit, where person-job was positioned as the exogenous variable, grit the mediator, age as covariate for grit, and subjective career success the endogenous (outcome) variable, is depicted in Figure 4.

The model fit indices suggested a model with acceptable fit (*χ*2 [3, *N* = 407]; = 3.82, *p* = 0.05; CFI = 0.99; TLI = 0.94; RMSEA = 0.08 [0.0–0.18]; SRMR = 0.02) The results indicated that grit significantly mediate the regression path from person-job fit to subjective career success (*β* = 0.09, *p* < 0.01, small effect). Age had a nonsignificant covariation effect on grit (*β* = −0.02, *p* > 0.01). Grit had a statistically significant direct effect on subjective career success (*β* = 0.41, *p* < 0.01, medium effect) and person-job fit had a statistically significant direct effect on subjective career success (*β* = 0.57, *p* < 0.01, large effect). The model explained 59% variance in subjective career success (*R*^2^ = 0.59). Hypothesis H5b was therefore confirmed. In the discussion to follow our findings of person-environment fit, refers to person-job fit and our grit findings refers to perseverance of effort.

## Discussion

As hypothesised (H1–H3), our results firstly confirmed the direct effect and positive influence of person-environment fit (i.e., person-job fit), resilience, and grit (i.e., perseverance of effort) on the subjective career success of women. Our results indicate that when women feel that their skills fit the demands of their job and their needs for rewards are being met (meaning they experience person-job fit in terms of need-supply fit and demand-ability fit), they value such fit to such an extent that increased feelings of subjective career success are evident. Ballout (2007) explains that because of the person-environment fit employees experience, they are more likely to engage in development-seeking behaviours and create situations that support higher job performance and achievement levels. Furthermore, our findings suggest that for women, having grit (specifically displaying perseverance and working hard towards set goals despite feedback) and having the capacity to bounce back from adverse situations in their careers or work environment adds to their experience of subjective career success. Noteworthy, 35% of our sample operated on middle and senior management levels in their organisations, which could relate to their objective career success experiences. Therefore, our findings align with previous research confirming that grit and resilience relate to employees’ career success (Bowman et al., 2015; Credé, 2018; Danner et al., 2020).

In addition to the direct effects of grit and resilience on subjective career success, our findings contribute significantly to the career literature of women by illustrating the moderating (the lack of) and mediating role of grit and resilience in the person-environment fit career relationship. From a JDR perspective, our study illustrates how the personal resources (i.e., grit and resilience) that women employ to adapt to their work environments influence their perceived career success. Furthermore, given that little is known about the moderating and mediating roles of grit and resilience, specifically in the person-environment fit and career success context, our study shed more light on how grit and resilience as personal resource can explain how person-environment fit (person-job fit) translates into subjective career success.

### The (lack of) moderating role of resilience

Although previous studies have found that resilience has a significant impact on the subjective career success of women (Wei and Taormina, 2014; Salisu et al., 2020), our results, unfortunately, could not confirm the moderating role of resilience in the relationship between person-environment fit and subjective career success. In this regard, we could not find support that women use resilience as a personal resource to support and strengthen the person-environment fit and subjective career success relationship. Furthermore, resilience as a personal resource did not act or provide a buffering effect in the case of poor person-environment fit for women concerning their experiences of subjective career success. Meaning women experiencing alignment between their personal characteristics, needs, and abilities are no more likely to experience subjective career success than those who report lower or higher levels of resilience. Such a finding suggests that the negative influence of poor person-environment fit on women’s perceptions of their career success can thus not be offset by activating their resilience or resilient behaviour. There could be several reasons for this. One reason for resilience not moderating the person-environment fit and career success relationship might be ascribed to the fact that women’s resilience will only become salient during challenging times. Resilience will thus not play a vital role when conditions in the working environment are easy and non-threatening and not a threat to women’s resources (Del Castillo, 2020), as may be in the case of good person-environment fit. This is also in line with the COR theory postulating that using personal resources such as resilience will play a more important role in challenging situations and when women have to use their resources to deal with challenges and avoid undesirable outcomes. Although various obstacles and barriers are found in the literature in which women are confronted, our findings suggest that only in the case of obstacles that threaten the person-environment fit women’s experience will require or activate resilient behaviour. In this regard, Caniels et al. (2022) argued that for individuals to behave resiliently, they need to activate the resilience trait. In this sense, Caniels et al. (2022) contend that resilient behaviour is not triggered in trait-resilient individuals when they do not perceive the organisational climate as error-avoiding (related to person-environment fit.).

### The mediating role of resilience

Although no moderating effect of resilience was found in our results, our findings did illustrate resilience as an explanatory variable in the person-environment fit career success relationship. Although a direct relationship between person-environment fit and career success has been found, resilience as a mediator can explain how person-environment fit translates into subjective career success for women. In this sense, Caniels et al. (2022) explain how good person-environment fit result in resilient behaviour. Their results indicated that an appreciation learning climate and a facilitating learning climate (which can be considered good fit) mediate the relationship between trait resilience and resilient behavior at work. In this sense, because trait-resilient women experience their working climate as positive (person-environment fit), they illustrate more resilient behaviour and are more likely to endure or bounce back from other obstacles or adversities in their careers and, as a result, still experience subjective career success. Salisu et al. (2020) also illustrated the mediating role of resilience and how women entrepreneurs are resilient to hardships and, as a result, keep on achieving their career goals and are more likely to succeed in their careers. Mishra and Mcdonald (2017) synthesized empirical literature on career resilience within the context of careers. They explained how *career* resilience is not a one-time event but rather a process that unfolds over a person’s career and how employees develop skills over a period of time. In this regard, the mediating role of career resilience between antecedents and career outcomes has been explored, where Chiaburu et al. (2006) contended that career resilience “is an important component in focusing proactive behaviors, because it brings together the necessary long-term commitment and persistence needed to engage in career self-management” (p. 623). Although we did not measure career resilience *per se*, our findings in the context of more contemporary definitions of careers and career success seem to align very much with the aforementioned mediating role of career resilience. Overall, it thus seems that women use their resilience capability to support them in overcoming precarious situations and even rise from let-downs and predicaments sturdier than before, making them more likely to succeed in their career pursuits (Han et al., 2021).

### The (lack of) moderating role of grit

Our findings indicate that grit did not exhibit a significant moderating effect on the relationship between person-environment fit and subjective career success. Our hypothesis is thus not supported. This is line with recent studies that also could not confirm the moderating effect of grit as a personal resource for instance in the relationship between person-environment fit and task performance (Van Zyl et al., 2022). Although Ma et al. (2020) confirmed that grit’s consistency of interest component moderated the association between teacher autonomy support and social competence, the perseverance of effort component did not. Our results seem to align with the results found in these previous studies confirming that higher perseverance levels of grit amongst women will not necessarily assist them in buffering the negative influence that poor environment fit will have on their subjective career success. More specifically, women will thus not use their grit as a personal resource to compensate for their perceived lack of fit, which could negatively influence their career success perceptions (Appel-Meulenbroek et al., 2019). Thus, it seems that women will not necessarily activate grit to deal with mis-fit and avoid it having a negative impact on their careers or to strengthen the person-environment fit and subjective career success relationship.

### The mediating role of grit

Our results show that the perseverance component of grit mediates the relationship between person-job fit and subjective career success and age as control variable. The inclusion of age did not significantly influence the mediation results. The mediating role of grit as a personal resource between variables is supported by previous research. Within the motivational process of the JDR model, Xanthopoulou et al. (2007) established that personal resources (such as grit and resilience in our study) mediated the relationship between job resources (i.e., personal-environment fit) and performance outcomes (such as subjective career success in our study). Similarly, the mediating role of grit in the relationship between person-environment fit and task performance was confirmed by Van Zyl et al. (2022) in their study. According to our results, women who perceive a good ‘fit’ between their skills and the rewards they can receive as part of their job will use their perseverance and strength to achieve their career-related goals despite obstacles and setbacks they might face. Our results are supported by the theory of Psychological Strengths (Peterson and Seligman, 2004) that in the presence of good person-environment fit, women will more readily live out their strengths (i.e., grit), which will help increase their career success perceptions. Our study has confirmed that the perseverance component of grit seems to be an essential mechanism in explaining how women’s person-job fit may translate into positive perceptions of the success of their careers.

## Theoretical contributions

Research on women’s career success predominantly outlines the many barriers/obstacles they face during their career paths. Despite an increased scholarly interest in positive psychology and growth development, far less consideration has been given to how women strengthen or use their internal resources to sustain their career goals and to, despite all odds, experience career success. Against this backdrop, our findings contribute to the career literature on women by providing evidence of how person-environment fit, grit, and resilience can be linked to women’s perceptions of their career success. Secondly, we answer the call of Su et al. (2015) for more research toward understanding the mechanisms (or psychological processes) and factors underlying the fit and fit-outcome relationships. In this regard, our findings illustrate subjective career success as an outcome of person-job fit and provide valuable insights on how personal resources (i.e., resilience and perseverance of effort) as part of the motivational process in the JDR-model can be used to explain the subjective career success experiences of women. Our findings provide more conclusive evidence of the mechanisms (or psychological processes) and factors underlying the person-environment fit and subjective career success relationship.

Concerning the role of resilience in the career context of women, finding evidence for its mediating role and not moderating role in the person-environment fit career success is quite significant. Although the mediating role of *career* resilience has been explored, providing evidence for resilience within the career context is new. It adds to ongoing debate on the difference between career resilience and resilience (Mishra and McDonald, 2017). Furthermore, similar to other studies, our study could not confirm the moderating role of grit but found evidence for the role of grit as a mediator in the person-job fit and subjective career success relationship. Our study advances our understanding of the person-environment fit and subjective career success and the valuable role that grit can play in the motivational process of the JDR-model.

## Implications of the research

Our findings have demonstrated that both resilience and grit (i.e., perseverance of effort) can play two distinct roles in the relationship between person-environment fit and subjective career success for women: direct and mediating. Focusing on the mediating role, resilience and grit appear to be malleable and influenced positively by their experiences of person-job fit. One obvious implication is that if women experience good job-fit there is greater opportunity for developing resilience and grit and, consequently, subjective career success. Therefore, improving women’s person-job fit is important to develop their resilience and grit. In this regard, the role of managers in facilitating the person-job fit of women is crucial. Women need opportunities to use their skills in their job and feel they contribute to the outcomes of their jobs. Consideration could also be given to matching the specific skills of women to specific jobs to obtain fit. Obtaining job-fit (or complimentary fit) may result in more positive experiences and opportunities for using their grit and resilience strengths. In line with Jogulu and Franken (2022), we recommend that women more effectively utilise resources within their job to build resilience and grit by emphasising the role of women’s network leveraging, learning, and adaptability. In this regard, women’s effective use of networking and mentoring opportunities and the positive relationship with their career success has been well documented.

## Limitations and suggestions for future research

The study had several limitations. First, this was a cross-sectional study, and self-report data were used, prone to common method variance (CMV) and social desirability, which might artificially augment the observed associations. Despite our efforts to reduce CMV by making use of different scale formats as suggested by Rindfleisch et al. (2008) and using familiar constructs to ensure that the wording of the questions was clear and concise (Rodríguez-Ardura and Meseguer-Artola, 2020), we cannot guarantee the causality of our results. We suggest that longitudinal data be obtained to understand better the causal influences among the variables (Ployhart and Vandenberg, 2010). There are contradictory findings in the literature regarding the influence of age on grit. Some studies (Pena and Duckworth, 2018) have shown that grit increases with age, suggesting that grit scores are malleable and can change over time. However, similar to our results, other studies (Duckworth et al., 2007; Clark and Clark, 2019) have shown no relationship between age and grit. Thus, researchers should be cautious about the impact of age when using a cross-sectional design since it might not account for potential influences of maturational processes on personality dimensions, especially on grit.

Several studies have debated the factor structure of the Grit-S scale as either a two-factor structure consisting of both perseverance of effort and consistency of interest component (Li et al., 2018; Schmidt et al., 2018; Van Zyl et al., 2022) or as a unidimensional construct (Ion et al., 2017; Gonzalez et al., 2019). We had to abandon the consistency of interest dimension in our study because it seems that the negatively scored items of this scale gave rise to problems in interpreting the items. Previous research (Bowman et al., 2015; Credé et al., 2017; Danner et al., 2020) concurred that the key function of the grit construct in predicting success rests in the perseverance of effort component. However, other researchers, such as Jachimowicz et al. (2018), emphasised the importance of passion (interest) as a critical component of grit in predicting performance. They suggested that this component be adequately measured to uncover grit’s true predictive power. Therefore, researchers are advised to use appropriate grit measures that match its definition.

## Conclusion

Overall, our findings illustrate the vital role of resilience and grit in the subjective career success of women by providing conclusive evidence these resources play indirectly in the person-environment fit and personal career success relationship of women. Using the motivational process of the Job Demands Resources Framework as theoretical background, we contribute by shedding light on how personal resources (resilience and grit) can be considered the underlying factors that influence the person-environment fit and career success relationship for women. If women experience good person-environment fit, there is a greater opportunity for developing resilience and grit and, consequently, subjective career success. Overall, organisations are advised to pay more attention to the person-job fit women experience as a means to build their resilience and grit capacity, which induce feelings of subjective careers success.

## Data availability statement

The raw data supporting the conclusions of this article will be made available by the authors, without undue reservation.

## Ethics statement

The studies involving human participants were reviewed and approved by the Department of Human Resource Management ethics committee, University of Pretoria. The patients/participants provided their written informed consent to participate in this study.

## Author contributions

EK and CO conceptualized the research and wrote the majority of the manuscript. PS assisted in the statistical analyses and reporting thereof. All authors contributed to the article and approved the submitted version.

## Conflict of interest

The authors declare that the research was conducted in the absence of any commercial or financial relationships that could be construed as a potential conflict of interest.

## Publisher’s note

All claims expressed in this article are solely those of the authors and do not necessarily represent those of their affiliated organizations, or those of the publisher, the editors and the reviewers. Any product that may be evaluated in this article, or claim that may be made by its manufacturer, is not guaranteed or endorsed by the publisher.
